# Fatty Acid Production and Direct Acyl Transfer through Polar Lipids Control TAG Biosynthesis during Nitrogen Deprivation in the Halotolerant Alga *Dunaliella tertiolecta*

**DOI:** 10.3390/md19070368

**Published:** 2021-06-25

**Authors:** Omri Avidan, Sergey Malitsky, Uri Pick

**Affiliations:** 1 Department of Metabolic Networks, Max Planck Institute of Molecular Plant Physiology, 14476 Potsdam-Golm, Germany; 2 Department of Plant and Environmental Sciences, The Weizmann Institute of Science, Rehovot 76100, Israel; sergey.malitsky@weizmann.ac.il; 3 Department of Biological Chemistry, The Weizmann Institute of Science, Rehovot 76100, Israel; uri.pick@weizmann.ac.il

**Keywords:** fatty acid turnover, polar lipids, oleic acid, polyunsaturated fatty acid, phosphatidylcholine, digalactosyldiacylglycerol, triacylglycerol biosynthesis, *Dunaliella tertiolecta*

## Abstract

The aims of this work were to evaluate the contribution of the free fatty acid (FA) pool to triacylglyceride (TAG) biosynthesis and to try to characterize the mechanism by which FA are assimilated into TAG in the green alga *Dunaliella tertiolecta*. A time-resolved lipidomic analysis showed that nitrogen (N) deprivation induces a redistribution of total lipidome, particularly of free FA and major polar lipid (PL), in parallel to enhanced accumulation of polyunsaturated TAG. The steady-state concentration of the FA pool, measured by prolonged ^14^C-bicarbonate pre-labeling, showed that N deprivation induced a 50% decrease in total FA level within the first 24 h and up to 85% after 96 h. The abundance of oleic acid increased from 50 to 70% of total free FA while polyunsaturated FA (PUFA) disappeared under N deprivation. The FA flux, measured by the rate of incorporation of ^14^C-palmitic acid (PlA), suggests partial suppression of phosphatidylcholine (PC) acyl editing and an enhanced turnover of the FA pool and of total digalactosyl-diacylglycerol (DGDG) during N deprivation. Taken together, these results imply that FA biosynthesis is a major rate-controlling stage in TAG biosynthesis in *D. tertiolecta* and that acyl transfer through PL such as PC and DGDG is the major FA assimilation pathway into TAG in that alga and possibly in other green microalgae. Increasing the availability of FA could lead to enhanced TAG biosynthesis and to improved production of high-value products from microalgae.

## 1. Introduction

Many green microalgae accumulate large amounts of triacylglycerides (TAG), amounting to 15–60% of their dry weight, under adverse environmental conditions such as nutrient deprivation and high light [1,2,3]. Because of the potential utilization of TAG for the production of biodiesel, the commercialization of algae has become of great interest [4,5,6,7,8,9]. The extensive research that has been done in the field in recent years has led to a better understanding of the regulatory mechanisms controlling lipid overproduction and accumulation in photosynthetic cells. A widely utilized approach to induce TAG accumulation in microalgae is to subject cultures to nitrogen deprivation. Such growth conditions partly uncouple the carbon-to-nitrogen (C/N) ratio, leading to attenuated chloroplast-cytosol-mitochondria communication, inducing photo-oxidative stress due to the accumulation of reducing equivalents and superoxide, all of which eventually result in the cessation of growth [10]. It has long been argued that N deprivation induces TAG accumulation as a protective mechanism against over-energization because its biosynthesis consumes large amounts of reducing equivalents. The synthesis of each C-18 FA requires 24 NADPH molecules, therefore de novo TAG biosynthesis could serve as an ultimate electron sink under photo-oxidative stress conditions [5]. 

A potential approach to enhance TAG production is to identify and upregulate rate-controlling key biosynthetic enzymes in the TAG biosynthesis pathway. According to current knowledge, TAG biosynthesis in higher plants and in microalgae proceeds in two consecutive stages: synthesis of fatty acids (FA), which takes place mostly within the chloroplast, and incorporation of FA into TAG, which in plants occurs in the endoplasmatic reticulum (ER), whereas in green microalgae, it takes place in both the ER compartment and in the chloroplast [10,11,12,13,14,15]. In plants, free FA (FFA) has been shown to play a signaling role in response to stress (e.g., infection), primarily by being oxidized and converted into downstream effector molecules such as bioactive oxylipins [16]. Cellular FA could originate either from de novo synthesis of acetyl-CoA or from the degradation of membrane lipids. The concentration of FFA is usually very low except in some diatoms [17], and most of the FA are bound either to CoA as acyl-CoA (in the cytosol), or to acyl carrier protein (ACP) as acyl-ACP (in the chloroplast). The acyl-CoA and the acyl-ACP pools are in constant equilibrium through the activity of the fatty acid export 1 transporter (FAX1) localized in the chloroplast envelope [18]. Because FA biosynthesis and the first two steps of FA/glycerol-3-phosphate esterification are common with polar glycerolipid biosynthesis, it may be expected that the rate-controlling step for TAG biosynthesis should reside in the terminal steps of the pathway/s, catalyzed either by diacylglycerol acyltransferase (DGAT) or by phospholipid:diacylglycerol acyltransferase (PDAT) through the acylation of diacylglycerol (DAG) by either free acyl-CoA or phosphatidylcholine-derived (PC) acyl units (sn-2, acyl editing) [19,20]. Indeed, an unusually large number of DGAT and PDAT genes/enzymes have been identified in some microalgae and it has been demonstrated that under N deprivation, some of the corresponding genes and enzyme activities are upregulated prior to TAG accumulation [21,22,23,24]. In addition, decreasing the activity of these enzymes by either RNAi and/or using knockout mutants has negatively affected TAG production. Therefore, it is widely accepted that these enzymes are among the major rate-limiting steps of TAG biosynthesis in algae and in plants [25,26]. Interestingly, attempts to increase TAG biosynthesis by overexpressing one of these enzymes resulted in conflicting results [27,28,29], suggesting a more complex regulation. 

To date, there are several indications that carbon availability, primarily at the early stages of the biosynthesis pathway, may also limit the rate of TAG accumulation: (i) Carbon flux measurements have indicated that in most oil-accumulating plants, the rate-limiting steps of TAG biosynthesis may reside not only within FA esterification into TAG, but also earlier in the FA biosynthesis pathway [30,31,32]; (ii) Supplementation of external carbon such as acetate or FA can boost neutral lipid accumulation in stressed algae, especially when applied mixotrophically; and (iii) Inhibition of the glyoxylate cycle and gluconeogenesis activities in N-deprived *Chlamydomonas reinhardtii* led to higher TAG content, potentially through increasing the availability of acetyl-CoA for FA biosynthesis [21,33]. Conversely, a comparative time-resolved transcriptome analysis between *Chlamydomonas reinhardtii* starch-less (sta6) and a few complemented mutants revealed conflicting results: N deprivation led to the upregulation of enzymes involved in the glyoxylate cycle and in gluconeogenesis in the high TAG sta6 mutant, possibly through increasing the availability of reducing equivalents needed to TAG biosynthesis [34,35]. Taken together, these results indicate that redirecting carbon supply into FA biosynthesis may be a major rate limitation in TAG accumulation [21,36,37].

We have recently shown that the rate of acetyl-CoA biosynthesis, the earliest committed substrate in FA biosynthesis, may play a major role in controlling the rate of TAG biosynthesis, particularly in oleaginous algae species [15,38]. In addition, we showed that most of the carbon utilized for TAG production in the high-starch low-TAG alga *D. tertiolecta* originates from starch and partially from directly assimilated CO_2_, and that these processes involve FA transfer through PL [15]. In this work, we tried to clarify to what extent the availability of FA and of different PL intermediates contributed to TAG biosynthesis during N deprivation in *D. tertiolecta* by kinetic and lipidomic approaches. For this, we estimated the changes in steady-state concentrations and in FA fluxes using ^14^C-bicarbonate labeling and ^14^C-palmitic acid (^14^C-PlA) incorporation into different lipid pools, respectively. We found that in the N-replete medium, incorporation of PlA into PC was faster than into any other lipid, indicating an active PC acyl editing, whereas under N deprivation, the flux through PC is attenuated and the flux through DGDG is enhanced. Furthermore, we used total lipidome analysis to identify early and late responses to N deprivation in both polar and neutral lipids. We found supporting evidence for acyl transfer through PL being the major FA assimilation pathway into TAG. Finally, we observed dynamic changes in the levels and composition of the FA pool and provide kinetic indications that the formation of FA and their utilization are accelerated upon N deprivation. The results suggest that the availability of FA, rather than their incorporation rate, is a major rate-controlling stage in TAG biosynthesis in *D. tertiolecta*.

## 2. Results

### 2.1. Changes in Lipidome Profile during Nitrogen Deprivation

Characterization of the dynamic changes in lipidome composition during N deprivation in *D. tertiolecta* was carried out by following the changes in the steady-state levels of selected lipid classes performed by a LC-MS based total lipidome analysis and by ^14^C-bicarbonate total labeling experiments.

#### 2.1.1. Lipidome Analysis

**Figure 1:**
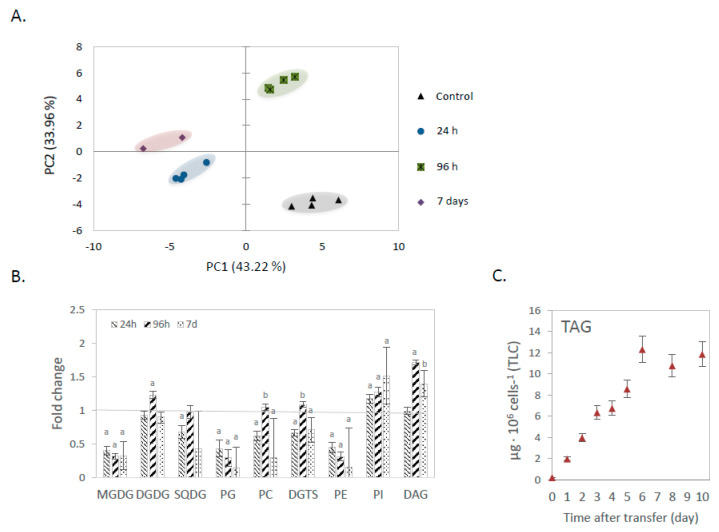
Changes in lipid composition following transfer to N deprivation measured by LC-MS. (**A**) Separation of major polar lipids (PL) by PCA. PCA1 and PCA2 explained 77% of the distribution of the data, and demonstrated a clear separation between the control and N-deprived cells as well as between early (24 h) and late (96 h, 7 days) time points. (**B**) Relative changes in the abundance of major phospholipid (PL) classes, presented as fold-change relative to control growth conditions. Error bars represent coefficient of variation. (**C**) Total triacylglycerol content (TAG) (µg 10^6^·cells^−1^, quantification by iodine stained band intensity, taken from Figure 2C). Error bars represent standard variation. Letters—statistically significant changes relative to control-tests done separately for each class using ANOVA, post-hoc, LSD test *p* < 0.05, with 4 independent replicates (apart from 7 days point, which is depicted by duplicates).

**Figure 2:**
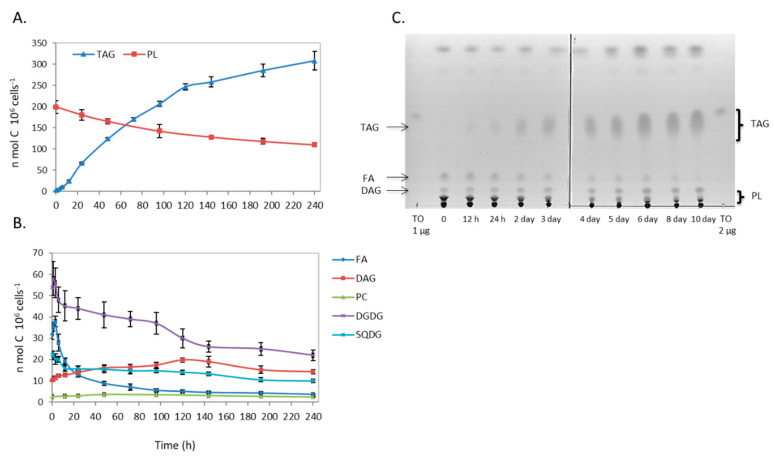
Changes in the steady-state levels of TAG and total PL (**A**), and of individual lipid classes (**B**), measured by total ^14^C labeling during N deprivation. *D. tertiolecta* cells were pre-cultured for 48 h in the presence of 50 mM ^14^C-bicarbonate and then transferred to deprived medium containing the same ^14^C bicarbonate concentration for 10 days. Samples were withdrawn at the indicated times, lipids were extracted, and separated on TLC plates (**C**) by n-hexane:diethylether:acetic acid (85:22:1), stained with iodine vapor, extracted, and ^14^C contents were determined. Each sample represents an extract of 10^6^ cells, error bars represent standard deviation (*n* = 3). TO—triolein TAG standard, PL—total polar lipids.

In order to obtain a complete profile of the changes occurring in different PL and TAG classes and of specific molecular species during N deprivation, we extracted lipids during different stages of N deprivation and analyzed their levels and compositions using LC-MS (described in the Materials and Methods). Equal amounts of cells were taken for analysis at the onset and after one, four, and seven days of N deprivation. These time points were selected to represent the first stage of N deprivation, the peak of TAG synthesis rate, and the end of the second stage (Figure 1 and Figure 2). The data were normalized to pre-spiked standards and analyzed with reference to a lipidome database created at the Weizmann Institute of Science (Rehovot, Israel), as described earlier [39]. 

A principal component analysis (PCA) of normalized peak area of all lipid species detected showed a clear discrimination between the control and N-deprived samples, mainly through PC1 (Figure S1), indicating redistribution of the lipidome during acclimation to N deprivation. In order to eliminate the contribution of TAG, which clearly differentiates between the control and N deprived cells, we applied a second PCA of major PL components alone and excluded TAG (Figure 1A). Similarly, the PCA explained 77% of the distribution of the data and showed a clear separation between all sample groups through both components (PC1, PC2), implying that these cells undergo remarkable lipidome remodeling, accompanying the progressive accumulation of TAG. Figure 1B summarizes the changes in the levels of major PL classes over time, showing an obvious decrease in most PL classes, in agreement with previous results in other unicellular algae [9,10,40,41,42]. Total MGDG, phosphatidylglycerol (PG), and phosphatidylethanolamine (PE) levels dropped by at least 50% within 24 h and stayed low for seven days, while total DGDG levels were maintained and remained comparable to the control at all time-points. Sulfoquinovosyl diacylglycerol (SQDG), PC, and diacylglyceryltrimethylhomo-Ser (DGTS) decreased to a smaller extent, reaching 30% to 70% of control after seven days. In contrast, total phosphatidylinositol (PI) and DAG increased in levels by up to 1.5-fold. These observations were mostly in line with previous studies in other green algae [11,40,43]. 

One intriguing observation is the small increase in total levels of DGDG, SQDG, PC, DGTS, and DAG after 96 h. Such an increase may reflect a late acclimation response in order to either maintain membrane integrity or support TAG biosynthesis, which is nearly at its maximal levels at this time point. A more comprehensive analyses of the changes occurring in specific lipid molecular species within each class is depicted in Figure S6 and is further elaborated in the Discussion. In terms of TAG, *D. tertiolecta* cells contain very low amounts of TAG when grown in complete growth medium (a total of 0.2 µg per 10^6^ cells, which represent approximately 2% of total lipids) (Figure 1C and Figure S4B). Following seven days of N deprivation, total TAG levels reached 12 µg per 10^6^ cells, corresponding to a 60-fold increase (relative to control), and constituting about 15% of total cellular carbon (Figure 1B; also discussed in [15]). Similar to other green algae species, the 50, 52, and 54 carbon TAG forms predominate, constituting approximately 90% of the total detected TAG (Figure S4A). In addition, there were few notable changes in TAG composition following N deprivation (Figure S4B): An increase in the relative abundance of most polyunsaturated TAG molecular species (50:3–50:7, 52:4–52:7, Figure S4B), a nearly complete disappearance of short-chain TAG 46C, and a remarkable drop in the relative levels of TAG 52:9, which was the predominant form under control conditions but dropped from 18% to 4% of total TAG during N starvation (total level increase, Figure S4B lower panel). The significance of these findings will be discussed below.

#### 2.1.2. Total ^14^C Labeling

In parallel to the LC-MS analysis, we used a ^14^C-bicarbonate labeling approach to estimate the steady state concentrations and pool sizes of FA and of various PL classes. For that, *D. tertiolecta* cultures were incubated with ^14^C-bicarbonate in a complete growth medium for 48 h. Under these conditions, cell number and biomass increased by a factor of ×50 and thus it can be assumed that all active carbon pools have been homogenously labeled with ^14^C (see Supplementary File 1). 

After 48 h in complete growth medium, the cultures were transferred to N-deficient medium containing the same concentration of ^14^C-bicarbonate and the incubation was continued for another 10 days, conditions that induce maximal TAG accumulation in *D. tertiolecta* (Figure 2A). Samples were withdrawn at different stages, lipids were separated by TLC, and then extracted and ^14^C contents were measured. The calculated levels of TAG, total PL, and of different lipids are shown in Figure 2A,B. TLC resolution of neutral lipids is shown in Figure 2C (PL were resolved using a different running solvent system, as previously described in [15]). In line with the lipidome data, ^14^C-bicarbonate labeling showed that during the progress of N deprivation, there was a marked increase in TAG synthesis and a decrease in total PL (Figure 2A, also in [15,44]) and that the accumulation of TAG is not linear with time but starts after a lag of about 12 h (Figure 2A,C). 

In terms of specific PL, we were able to separate the following lipids with high confidence: SQDG, DAG, PC, FFA, and DGDG (see Figure S1 in [15]). The changes in levels of the most examined PL corresponded fairly well with the lipidomic data, except for DGDG: the total SQDG level slightly decreased during the first 24 h of N deprivation, while total PC level remained initially stable, peaked after 96 h, and then gradually decreased. It should be noted that total PC level was significantly lower than that of most other PL. Total DAG increased by 1.8-fold after five days and thereafter decreased. 

The FA pool, which could not be detected under the conditions used for the LC-MS, was slightly increased during the first 1–3 h, followed by a sharp decrease of 60% after 24 h and by almost 80% after 48 h. The half time for the decrease in FA level was approximately 12 h (Figure 1B and Figure S2). The observed biphasic behavior indicates dynamic changes in the rates of FA production and/or utilization, which may be related to the lag observed in TAG production, as will be discussed below. The discrepancy between the apparent decrease in DGDG pool size measured by total ^14^C labeling (Figure 2B), and the stable levels observed by LC-MS lipidome data (Figure 1B and Figure S6) are puzzling. Possible explanations for this discrepancy may be the following. (1) A contamination of the DGDG spot on the TLC by MGDG, which sharply decreases (Figure 1B). Under the separation conditions applied, MGDG migrates close to DGDG (on TLC) and might be accidently collected [15]. The relatively stable DGDG levels observed in other species favors this explanation. (2) The lipidome data are lacking several prominent unidentified DGDG species that are responsible for the major decrease in pool size.

**Figure 3:**
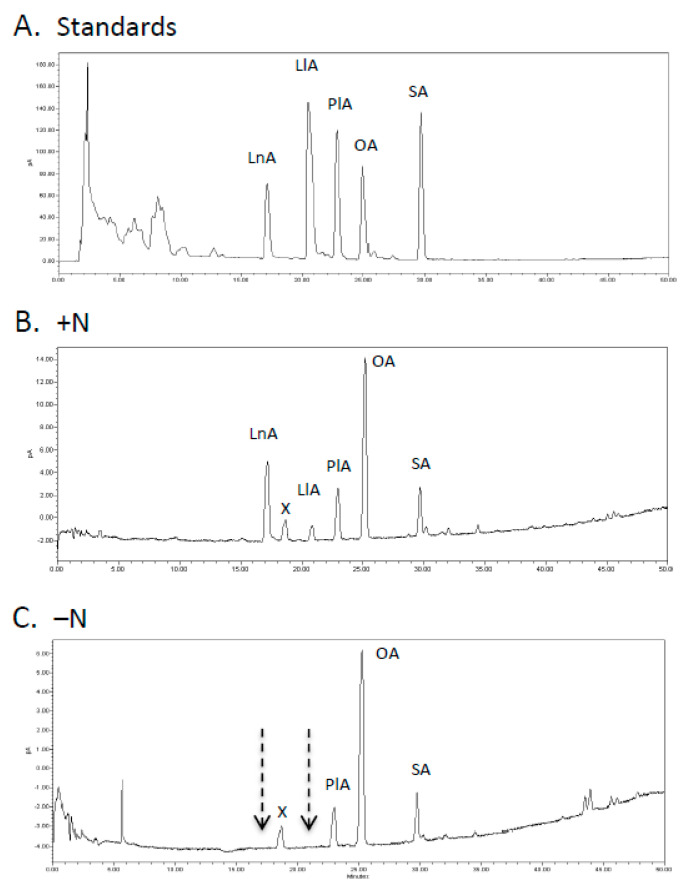
Free fatty acid compositions in the control and in N-deprived cells. *D. tertiolecta* cells were cultured for 2 days in complete growth medium (+N) or for 4 days in N-deficient medium (-N). Samples of 1.5 × 10^8^ cells of each culture were extracted, lipids were separated on TLC plates, FA bands were cut out, extracted, and analyzed on a Halo C8 HPLC column. (**A**) Mixture of FA standards, 2.5 μg each. (**B**) control cells, (**C**) N-deprived cells. PlA–Palmitic acid (16:0), LnA—linolenic acid (18:3), LlA—linoleic acid (18:2), OA—oleic acid (18:1), SA—stearic acid (18:0), X—not identified.

In order to estimate the internal FFA concentration, we designed a competition experiment between externally added PlA to the endogenous PlA pool, as explained in Figure S3B. The estimated internal free PlA concentration, acquired from the competition experiment, was around 1 μM. Since PlA constitutes around 10% of the FFA pool (Figure 3), the total FFA concentration was estimated to be around 10 μM. Furthermore, in order to learn if the FA pool changed in composition during N deprivation, the FA TLC bands were cut out, extracted, and analyzed by reverse-phase HPLC on a Halo C8 column against common FA standards. As shown in Figure 3, the FA bands contained a mixture of 4–6 major FA, with oleic acid (18:1) being the major component in both conditions (50–70%). Similar results have been reported previously for another *dunaliella* species [44,45,46]. The results are summarized in Table 1. The major differences in FA composition between the two extracts were the relative increase in levels of oleic acid and the complete absence of polyunsaturated FA, 18:2, and 18:3 in the N-deprived (96 h) cell extracts. This observation is particularly interesting in light of the concomitant increase in the relative abundance of polyunsaturated TAG (Figure S4B). A likely candidate for the unidentified X component may be 16:4, which comprises 5–15% of total FA in *dunaliella* [46]. The significance of these results to the mechanism of TAG biosynthesis will be discussed below.

### 2.2. Estimation of FA Fluxes through Different Lipid Pools Using ^14^C-PlA

In order to estimate and compare the rates of synthesis of different lipids under control and N-deprived conditions, we used pulse-labeling with ^14^C-PlA because it is rapidly and selectively incorporated into glycerolipids including TAG with very little incorporation into other cellular components [15,44,47]. The rapid incorporation of PlA into different lipids suggests that there are no diffusion barriers for the equilibration of PlA into different cellular compartments [15], and therefore the initial rates of PlA incorporation can be considered as a measure of FA flux. In order to test whether PlA supplementation affects the physiology or metabolism in *D. tertiolecta*, we measured the effects of increasing PlA concentrations on cell proliferation and on TAG accumulation (Figure S3A). At the labeling concentrations used here (0.5 μM), PlA addition does not have any significant effects, however, higher concentrations (10–100 μM) slightly increased TAG levels, consistent with previous studies [37].

**Figure 4:**
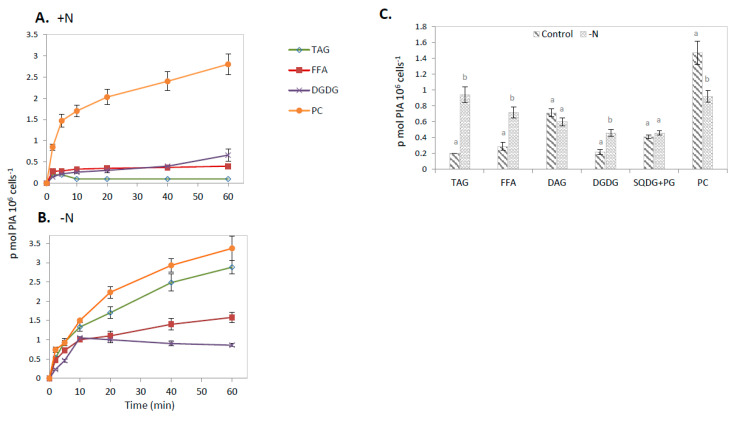
Time course of incorporation of ^14^C-PlA into different lipids. *D. tertiolecta* cells cultured for 48 h in either complete (**A**) or in N-deficient (**B**) media supplemented with ^14^C-PlA. Samples were withdrawn, quenched, and washed, lipids were extracted, separated on TLC, and analyzed as described under the Experimental Procedures. (**C**) comparison of +N and −N absolute incorporation values after 5′ of incubation. Values are expressed in p mol PlA 10^6^ ·cells^−1^ and represent averages of 3 experiments. Error bars represent standard deviation while letters represent statistical tests within each class separately using ANOVA, post-hoc LSD test, *p* < 0.05.

*Dunaliella* cells were cultured for 48 h either in N-replete (control) or in N-deficient media and were then supplemented with ^14^C-PlA (pulse) for 1 h. Samples were withdrawn after: 0, 2, 5, 10, 20, 40, and 60 min, extracted, and individual lipids were analyzed as described in the Materials and Methods. As shown in Figure 4A (and in Figure S5), the incorporation of PlA into different lipids was linear only within the first 2–10 min. Therefore, we chose the first 5 min to calculate the initial rates of incorporation. As summarized in Table 2, the incorporation of PlA into PC in complete medium was faster than into any other identified lipid and remained higher than other lipids at all time points during the first hour of labeling. All other PL were labelled more slowly, consistent with a dominant PC acyl editing mechanism under N-replete growth conditions. These results are remarkable in light of the low steady-state concentrations of PC in *D. tertiolecta* (Figure 2B, Table 2), but they are in agreement with previous studies in plants and in other algae [48,49,50]. Incorporation of PlA into the FA pool saturates within 2–5 min and remains low. 

Under nitrogen deprivation, there are significant changes in the rates of incorporation and saturation levels of several lipid classes. The incorporation into PC decreased by approximately 30%. In contrast, the rates of incorporation into DGDG and into the FA pool increased by 2 and 2.5-fold, respectively, in parallel with the decrease in their steady-state concentrations (by 15% and 70% during the first 48 h, respectively, Figure 2B). These observations suggest a rapid turnover of DGDG and of the FA pool under N deprivation. The rate of incorporation of PlA into SQDG + PG was not significantly affected by N deprivation, but their steady-state levels decreased, potentially reflecting the degradation of chloroplast membranes (consistent with the LC-MS data, see Figure 1B). Regarding DAG, there was a small decrease in the rate of incorporation into DAG at 48 h of N deprivation, while the steady-state levels increased significantly (by 1.5-fold), suggesting that most DAG species were probably not being incorporated directly into TAG, but rather integrated first into a PL intermediate. Interestingly, a more detailed examination of the relative changes occurring in the levels of specific DAG molecular species (Figure S6) revealed a radical change in the profile of abundance of DAG species during N deprivation whereas the major DAG molecular species 36:0 (which represents 35% of total DAG under control conditions) decreased by 60%, and polyunsaturated molecular species transiently increased, peaking at 96h of N deprivation. Finally, the incorporation rate of PlA into TAG showed the highest relative increase following 48 h of N deprivation and is in line with the increase in levels of TAG observed at this stage (Figure 2A). 

Taken together, these results suggest that during N deprivation, there was a partial inhibition in PC acyl editing, accompanied by the enhancement of carbon flow through DGDG and FA. These results highlight the possible involvement of FA and of DGDG in TAG biosynthesis, as will be discussed below.

## 3. Discussion

### 3.1. The Dynamics of the FA Pool

The FA composition observed in this work was dominated by oleic acid (18:1), consistent with previous reports in higher plants and algae [39,51,52]. Following 96 h of N deprivation, there was a large increase in the relative abundance of oleic acid and a complete disappearance of polyunsaturated FA (PUFA), 18:2, and 18:3. The increase in abundance of oleic acid is in line with previous studies, showing that this FA is a major constituent of TAG in green algae, and with the reported increased activity of an MGDG-specific lipase like Plastid Galactoglycerolipid Degradation1 (PGD1) (*C. reinhardtii*), which preferentially hydrolyzes oleic acid from special newly-formed molecular species of MGDG, to be eventually incorporated into the sn1/3 positions in TAG [43,53]. In contrast, the disappearance of the polyunsaturated FA, 18:2, and 18:3 is intriguing. Under N deprivation, chloroplast membrane lipid degradation results in the transfer of FA (including PUFA) into TAG, as we also previously demonstrated by the transfer of PlA from pre-formed membrane lipids in *D. tertiolecta* [15]. Therefore, if membrane lipid breakdown results in the release of FA directly into the FA pool, it should be expected that the levels of PUFA in the FA pool would rise and not decrease during N deprivation. Moreover, because desaturation of FA in plants and eukaryotic algae from monounsaturated FA onward takes place on membrane-bound lipids during the biosynthesis of MGDG and of DGDG in the chloroplast, or of PC in the ER [30,32,54], the PUFA in the FA pool should derive primarily from the degradation of pre-formed lipids and not from de novo synthesis. The absence of PUFA in the FA pool during N deprivation suggests, therefore, that the transfer of 18:2 and 18:3 from PL into TAG is mediated by the direct transfer of acyl chains without the release of free FA into the FA pool. 

Indeed, major candidates to mediate chloroplast membrane lipid hydrolysis under N deprivation are PLIP1, PGD1, and PES-like enzymes, which catalyze the exchange of acyl chains from chloroplast membrane lipids into TAG [55,56]. We have previously identified in *Dunaliella bardawil* three different PES proteins that are upregulated under N deprivation and could mediate such an exchange [57]. Acyl exchange between phospholipids and TAG in the ER such as mediated by PDAT has also been characterized and shown to be activated under N deprivation in plants and in algae and has also been identified in *dunaliella* [57,58]. Such acyl exchange mechanisms are energetically favorable compared to acyl hydrolysis, followed by re-esterification into acyl-CoA or acyl-ACP. The importance of these enzymes for TAG biosynthesis therefore implies that incorporation of FA into chloroplast glycerolipids is a major and possibly a prerequisite step for TAG assembly. 

### 3.2. Changes in the FA Pool Are Associated with TAG Biosynthesis

**Scheme 1:**
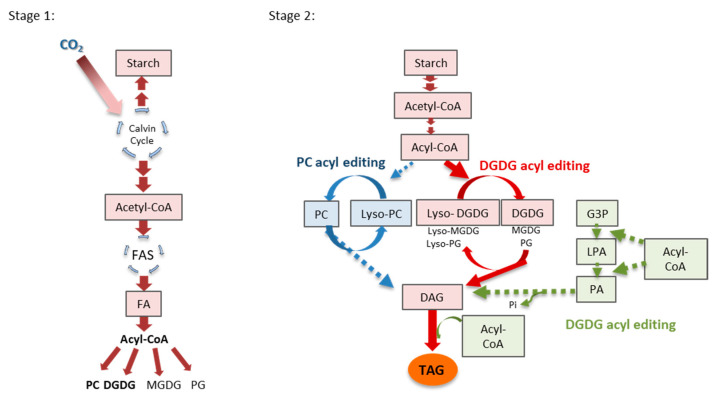
The proposed carbon flow leading to TAG biosynthesis in *D. tertiolecta*: distinction between early and late phases of N deprivation. In the early phase (stage 1), cells photo-assimilate CO_2_ primarily into starch. Part of the assimilated carbon is tunneled toward acetyl CoA production, resulting in activation of FA biosynthesis. In the late phase (stage 2), starch breakdown supplies carbon for FA biosynthesis. The produced acyl-CoA can be integrated into TAG either through the Kennedy pathway, through PC-acyl editing, or through DGDGacyl editing, which is activated under these conditions. Bold/Red indicates major induced pathways, Dashed/Blue/Green—minor/less active pathways. FA—fatty acids, PC—phosphatidylcholine, DGDG—digalactosyl-diacylglycerol, MGDG—monogalactosyl-diacylglycerol, PG—phosphatidylglycerol, DAG—diacylglycerol, G3P—glyceraldehyde 3-phosphate, LPA—lysophosphatidic acid, PA—Phosphatidic acid.

In a previous work, we have shown that the response of *D. tertiolecta* to N deprivation is biphasic: During the first 24 h, the cells mainly accumulate starch. From the second day, cell metabolism shifts toward TAG production by utilizing carbon originated mostly from starch degradation [15]. This hypothesis is further elaborated in Scheme 1. In the present work, we have characterized the changes in the lipidome of *D. tertiolecta* during N deprivation that presumably lead to TAG biosynthesis. We found that during the first 24 h, there are dynamic changes in the concentrations and turnover of the FA pool, which seem to be associated with the activation of TAG biosynthesis. In particular, we can see that during the first 3 h of N deprivation, the FA pool level slightly increased and then progressively declined, reaching 50% of the control after 24 h and 20% after 48 h. Similar observations were previously described for *Chlamydomonas reinhardtii* when grown mixotrophically and subjected to N deprivation conditions [33]. It has been postulated that *C. reinhardtii* cells respond rapidly by enhancing gluconeogenic metabolism during the first 4–6 h and then switch to a glycolytic stage, involving the activation of nitrogen recycling pathways and lipid synthesis. The increase in rate of PlA incorporation into the FA pool in *D. tertiolecta*, combined with the decrease in steady-state levels of FA during the early stages of N deprivation (Table 2), suggest a rapid activation of FA biosynthesis followed by enhanced FA utilization upon transfer to N-deprived conditions. A fast activation of FA biosynthesis is consistent with our previous observations of rapid increase in levels of acetyl-CoA, preceding TAG formation, in response to N deprivation in several green algae [38]. With regard to *D. tertiolecta*, we have calculated a 6-fold increase (at peak levels) in cellular acetyl-CoA concentrations already within 16 h of N deprivation. Moreover, a comparison between high vs. medium vs. low TAG accumulating algae revealed a close correlation between the cellular concentrations of acetyl-CoA and TAG accumulation capacity. These correlations strengthen the significance of the FA pool and of acetyl-CoA for TAG production and accumulation in green algae. The rapid changes of the FA pool size precede the major activation stage of TAG biosynthesis, which occurs between the first and second days of induction (Figure 1B and Figure 2C; see also [15]), and thus are consistent with the idea that the decrease in FA pool size may result from accelerated assimilation into TAG. These findings support our previous suggestion that production of acetyl-CoA may be the rate-limiting step in the activation of FA biosynthesis and consequently of TAG biosynthesis and accumulation. Most of the acetyl-CoA is eventually incorporated into PL such as PC and DGDG [15]. A summary of the major metabolic changes during the early stages of N deprivation is depicted in Scheme 1 (stage 1).

### 3.3. What Is the Biosynthetic Pathway Responsible for FA Integration into TAG?

A potential candidate for FA biosynthesis is the Kennedy pathway, a canonical de novo pathway of TAG biosynthesis that resides in the ER and involves DAG as a major intermediate [49]. Indeed, the 1.5–1.7 fold elevation in total DAG levels (Figure 1B and Figure 2B), combined with the remarkable changes in DAG molecular species composition (decrease in the major 36:0 level and increase in relative levels of 34:1, 34:2, 34:3, 36:2, 36:4; Figure S6), suggest that the Kennedy pathway is activated. However, this is inconsistent with the finding that the flux of FA into DAG does not increase but rather slightly decreases following 48 h of N deprivation (Table 2). The significance of these results is uncertain since DAG is also involved in the biosynthesis of most polar glycerolipids such as MGDG, PC, and SQDG, which are mostly inhibited under N deprivation (Figure 1B; [15]). This complicates the interpretation of rates of PlA incorporation into DAG [3,30,48,49]. In that respect, a distinction should be made between de novo synthesized and PC-derived DAG. As noted above, the Kennedy pathway is responsible for assimilating de novo synthesized FA into a glycerol backbone to generate TAG, involving DAG as a major intermediate. However, this process may also involve FA exchange with PC, namely, PC acyl editing, leading to the generation of PC-derived DAG [50]. Increase in the level of PC-derived DAG (or other PL-derived DAG) will delay the incorporation of ^14^PlA into DAG and thus may underestimate FA-to-DAG values.

The second major candidate pathway for incorporation of FA into TAG is PC acyl editing. PC acyl editing is the major pathway of FA desaturation and incorporation into PL in both higher plants and in microalgae [51,56], and has also been reported to be a major contributor for TAG biosynthesis [50,59,60]. Indeed, the rapid assimilation of PlA into PC, exceeding the incorporation into all other tested lipids (Figure 4A), indicates that in *D. tertiolecta*, PC acyl editing is also a major pathway for FA assimilation, at least under nitrogen-replete growth conditions. This observation is quite striking in light of the low steady-state levels of PC in *D. tertiolecta* (Figure 1B, Table 2), suggesting a much faster turnover of PC compared to other lipids. This may vary between species, specifically in those lacking PC such as *C. reinhardtii* [61]. Under N deprivation, the FA flux through PC decreased by 35–40% (Figure 4B, Table 2), but was still the highest rate of incorporation amongst all examined PL (similar to TAG). This observation, combined with the clear decrease in levels of almost all PC molecular species (Figure 2C and Figure S6), suggests that PC acyl editing becomes less active under N deprivation. 

### 3.4. Are There Alternative Acyl-Editing Pathways in D. tertiolecta?

The lipidomic data show different changes in response to N deprivation in the eight polar lipid classes that were examined (Figure S6). Comparing the two major cellular lipids, the chloroplast galactolipids MGDG and DGDG, revealed that whereas in MGDG the major molecular species (34:5, 34:6) drastically decreased, in DGDG, most of the molecular species hardly changed. These results are in contrast to a lipidomic study in *chlorella* and in *nannochloropsis* in which both MGDG and DGDG were found to decrease to a similar extent under N deprivation [40], but are consistent with other studies in *C. reinhardtii* and in *D. tertiolecta*, showing that the DGDG to MGDG ratio, an important determinant of lipid bi-layer integrity, increases upon stress [62,63]. 

In the present work, we found that the total level of MGDG decreased by about 50% during N deprivation, similar to previous studies in other green microalgae [40,62,63]. This decrease probably resulted from the degradation of thylakoid membranes, in which MGDG is the most abundant lipid, comprising 50% of the chloroplast membrane lipids. Several reports have suggested that degradation of MGDG may provide substantial amounts of FA for TAG biosynthesis under N deprivation. For example, studies in *C. reinhardtii* have identified a MGDG-specific lipase, PGD1, which mediates the release of specific FA from sn1 of MGDG, preferentially under stress conditions such as N deprivation [43,53,56]. In line with these results, we detected substantial amounts of lyso-MGDG 16:3, which gradually decreased over time, concomitant with an increase in lyso-MGDG 16:1 (Figure S6), both of which could be generated by a PGD1-like enzyme. The increase in MGDG 34:1, 34:2, 34:3 could thus be explained by a progressive degradation of the major MGDG molecular species, 34:5 and 34:6. Taken together with the observed increase in levels of polyunsaturated TAG molecular species during N deprivation (50:3–50:7, 52:4–52:8, Figure S4B), these results may indicate the transfer of polyunsaturated FA from MGDG into TAG during N deprivation. 

In contrast to MGDG, most of the molecular species of DGDG showed little changes in levels during N deprivation. However, a close examination showed that some polyunsaturated molecular species (34:3, 34:4, 34:5, 36:6) slightly decreased, particularly after seven days of N deprivation (Figure S6). In comparison to other PL, DGDG seems to be unique in several aspects: (i) it is the only PL whose levels are maintained throughout N deprivation without large changes in composition; (ii) it is also the only PL whose turnover rate seems to be significantly increased during N deprivation (Table 2); and (iii) in a previous study, we showed that de novo synthesis of DGDG continued during N deprivation, albeit at a reduced rate, whereas synthesis of all other measured PL was almost completely suppressed under the same conditions [15]. These results suggest that DGDG undergoes dynamic metabolic changes during N deprivation in *D. tertiolecta* and may have a special metabolic role in TAG biosynthesis. In our previous study, we showed that a substantial amount of TAG originated from newly synthesized FA that were pre-integrated into PL at early stages of N deprivation, prior to the incorporation into TAG [15]. Part of this acyl transfer reaction may be catalyzed by PC through PC acyl editing. However, the decrease in the incorporation of PlA into PC suggests that during N deprivation, PC acyl editing is partly suppressed. Therefore, we speculate that DGDG may be the alternative candidate to catalyze acyl transfer into newly synthesized TAG under N deprivation in *D. tertiolecta*. 

The level of the anionic phospholipid PG was found to decrease by about 50% during N deprivation. PG is the only major phospholipid in chloroplast thylakoid membranes, it is located in close proximity to the photosystems, and it has been shown to be indispensable for their function [63,64]. The major decrease in PG contents during N deprivation can be attributed to the 90% decrease in levels of PG 34:4, which represents 85% of total PG under normal growth conditions but only 15–25% following N deprivation (Figure S6). Therefore, the parallel increase of PG 34:1, 34:2, 34:3 that we observed is likely to be achieved by desaturation of PG 34:4 rather than by de novo synthesis. The accumulation of these PG molecular species could be associated with massive chlorophyll and thylakoid breakdown, and may serve a role in maintaining thylakoid membrane integrity under stress conditions. It is, however, interesting to relate these findings with a previous observation in seeds of *Arabidopsis thaliana*, in which a PG-specific lipase (PLIP1) was shown to mediate the release of 18:3 FA from the sn1 position of PG 34:4 and was suggested to be involved in subsequent acyl transfer into TAG [56]. It is therefore tempting to connect the decrease we observed in PG 34:4 to the increase in PUFA TAG, however, considering that PG constitutes about 10% of chloroplast membrane lipids, the degradation of pre-existing PG could only serve as a minor source of FA for TAG. Hence, we speculated that a PLIP1-like mechanism in *D. tertiolecta* could be involved in minor acyl editing and/or acyl transfer of newly synthesized FA into TAG.

Finally, it is interesting to compare the time-dependent kinetic response of different lipids following N starvation. A close examination of the lipidomic data revealed a common pattern of changes in levels of several lipids, particularly of major DGTS and PC species and most DAG, where all uniformly peaked at the 96 h point (Figure 1B and Figure S6). Considering that PC and DAG are the two key intermediates of the Kennedy pathway and of PC acyl editing that contribute to TAG biosynthesis, and that TAG accumulation was almost at its peak after 96 h, the common upregulation of these two lipids at this time point could therefore serve to provide FA intermediates to TAG synthesis, whereas the increase of DGTS may play a structural role in maintaining membrane integrity. 

### 3.5. The Origin of PUFA in TAG under N Starvation

*D. tertiolecta* is a moderate TAG accumulator and thus TAG is accumulated relatively slowly and to moderate levels (up to 20% DW, 10 µg per 10^6^ cells), reaching maxima after 5–7 days [15,38,65]. PUFA are major constituents of the FA composition in TAG in *D. tertiolecta*, and their relative levels increase during N deprivation (Figure S4B) [62]. These PUFA may originate either from degradation of chloroplast membrane galactolipids, particularly of MGDG, and partly through de novo synthesis involving a PL intermediate (PC and/or DGDG) [30,66]. In this respect, it is interesting to compare the time-resolved changes that we observed in the polyunsaturated molecular species of specific PL classes with the changes in profile of DAG species (Figure S6), and tried to correlate them with the biosynthesis of polyunsaturated TAG species (Figure S4). The DAG species that mostly increased were 34:1–34:3, 36:2, and 36:4, all of which peaked at 96 h of N deprivation. MGDG species that mostly increased were 34:2 (peaking at 24 h) and 34:3 (peaking at 96 h and 7 d). The only DGDG species that transiently increased in level was 34:6 (peaking at 96 h). PG species 34:1, 34:2, 34:3 transiently increased in level, peaking at 24 h of N deprivation. These results suggest that PG 34:1, 34:2, 34:3, and MGDG 34:2 serve in acyl transfer into TAG at the early stages of TAG biosynthesis; whereas MGDG 34:3 and DGDG 34:6 are the major contributors at 96 h, the peak of TAG biosynthesis, when the DAG 34:3, 36:2, and 36:4 species are also at their highest levels. Theoretically, DAG 34:3, could produce the major TAG molecular species 50:5, 50:7, 52:6 (by acylation of FA 16:2, 16:4, 18:3, respectively), which were indeed detected. These considerations, combined with the absence of free PUFA in the FA pool, support the idea that direct acyl transfer of PUFA, from PL to TAG, is the preferred mechanism for poly unsaturated TAG synthesis and it may significantly contribute to total FA assimilation under N deprivation in *D. tertiolecta*. The previously reported enhancement in the abundance of ER-plastid contact sites, generated by enlarged lipid droplets in *D. tertiolecta* [44], provide a spatial support for the existence of this mechanism and its potential contribution to TAG biosynthesis in this alga. 

## 4. Materials and Methods

### 4.1. Radioactive Chemicals

^14^C sodium bicarbonate (PerkinElmer, NEC086HOO, 53 mCi mmol^−1^) and ^14^C palmitic acid [^1−14^C] (PerkinElmer NEC075HO, 60 mCi mmol^−1^) were purchased from PerkinElmer, Waltham, MA, USA.

### 4.2. Algal Strains and Cultivation Conditions

*D. tertiolecta* was obtained from the culture collection of Dr. W. H. Thomas (La Jolla, CA). Cells were cultured under continuous illumination (at light intensity of 120–150 μE·m^−2^·s^−1^) on a shaker set at 100 rounds per min at 24 °C, as previously described [67], using 3–4 independent cultures. The media were supplemented with 0.5 M NaCl and 50 mM sodium bicarbonate, with initial cell concentration of 2 × 10^6^ cells mL^−1^. To induce N deprivation, mid-log phase cells (cultured for 48 h) were centrifuged for 5 min at 5000× *g*, washed once with growth medium lacking KNO_3_ (−N medium), and suspended in fresh −N medium. Cells were counted with the automated cell counter Cellometer (Nexelom Bioscience LLC, Lawrence, MA, USA).

### 4.3. Labeling with ^14^C-Bicabonate

*D. tertiolecta* were labeled with 50 mM ^14^C-bicabonate (0.16 μCi mL^−1^) in growth media containing 5 mM K-nitrate (+N) or no nitrate (−N). For total carbon labeling, cells were incubated with ^14^C-bicabonate for the whole growth period, namely, two days in +N and up to eight days in −N growth media. At the end of the incubations, cells were washed twice in fresh medium containing 50 mM bicarbonate to remove traces of ^14^C-bicabonate, flash-frozen, and stored at −20 °C for lipid extraction. Under these labeling conditions, the extent of the dilution of ^14^C-bicabonate by atmospheric CO_2_ was around 1% day-1 (Supplementary File 1) and thus ignored in our calculations. Included were three independent experiments (*n* = 3).

### 4.4. Labeling with ^14^C-PlA

*D. tertiolecta* cells were cultured for 48 h either in complete (+N) or in N-deficient (−N) growth media containing 0.5 M NaCl and 50 mM Na-bicarbonate. At time 0, the cultures were supplemented with ^14^C –PlA (1–2.5 μCi 100 mL^−1^, 0.5 μM; provided as an ethanolic solution) and incubated in an illuminated shaker. At the indicated time points, samples containing 3–12 × 10^7^ cells were taken out, washed once in a fresh medium containing 100 μM PlA at 4 °C, then incubated for 10 min in ice-cold growth medium containing 1% defatted bovine serum albumin (dfBSA) to eliminate all traces of ^14^C-PlA, washed once again in fresh growth medium, and the pelleted cells were stored at −20 °C for lipid extraction. These quenching conditions effectively stop the incorporation of ^14^C –PlA into lipids (Tables S1 and S2). Included were three independent experiments (*n* = 3).

### 4.5. Lipid Extraction and Analysis

Lipids were extracted essentially as described before [38]. In brief, cells were permeabilized by dimethylsulfoxide (DMSO) (5′ at 70 °C), pellets were extracted in MeOH:CHCl_3_:H_2_O, 1:1:1, the CHCl_3_ phase was concentrated and dissolved in 150 μL MeOH:CHCl_3_ 1:1 and kept at −20 °C for lipid analysis by thin layer chromatography (TLC) on silica gel 60 aluminum sheets (Merck, Darmstadt, Germany). Different quenching conditions were compared in order to verify that there was no significant hydrolysis of lipids during the quenching and extraction (Table S3). Neutral lipids, FA, diacylglycerol (DAG) and total PL were separated in n-hexane:diethylether:acetic acid (85:22:1) as shown in Figure 1C. Digalactosyl-diacylglycerol (DGDG), sulfoquinovosyl-diacylglycerol (SQDG), and phosphatidyl-choline (PC) were resolved either in chloroform:methanol:acetic acid:water (90:20:12:4) or in chloroform:methanol:water (60:30:4) as described in [15] Figure S1. Monogalactosyl-diacylglycerol (MGDG) was not analyzed for ^14^C because it was not well resolved. For determination of ^14^C content, plates were dried, stained with iodine vapor, and the desired bands were cut out, placed in a scintillation vial, supplemented with 1 mL CHCl_3_:MeOH, 1:1 and extracted on a shaker for 4–12 h. The extracts were transferred to a clean scintillation vial, supplemented with a scintillation cocktail, and counted in a scintillation β-counter.

Fatty acid HPLC analysis was performed according to [68] with a Halo C8 150 × 4.6 mm, 2.7 μm column, eluted with a gradient of the following mobile phases: (A) methanol:water:acetic acid (750:250:4) and (B) acetonitrile:methanol:tetrahydrofuran:acetic acid (500:375:125:4) according to the program: 0–46 min: 100–30A/0–70B; 46–60 min: 30–10A/70–90B; 60–65 min: 10A/90B; 65–65.1 min: 10–100A/90–0B; 65.1–72 min: 100A/0B. The column temperature was kept at 40 °C and the samples were cooled to 10 °C. The flow rate was 0.8 mL min^−1^. The lipids were detected using a Corona Charged Aerosol Detector (ESA Biosciences Inc., Chelmsford, MA, USA). FA was identified and quantified using FA standards.

### 4.6. Lipidomic LC-MS Analysis

Lipid extraction, detection, and analysis were performed as previously described in [39]. In brief: samples were collected periodically (four replicates) by filtration, quickly frozen by liquid nitrogen, and stored at −80 °C. For lipid extraction, samples were suspended in a methanol:methyl-tert-butyl-ether (TMBE) 1:3 (*v*/*v*) mixture containing 0.05–0.1 µg·mL^−1^ of standard mixture (of various PE, PC, TAG species). Organic phases were collected, dried under N_2_, and used later for analysis, as previously described. For lipid detection, samples were subjected to UPLC-MS analysis. Lipid extracts were analyzed using a Waters ACQUITY UPLC system coupled to a SYNAPT G2 HDMS mass spectrometer (Waters Corp., Milford, MA, USA). Chromatographic conditions were as described in Malitsky et al. (2016). LC-MS data were analyzed and processed with QuanLynx (Version 4.1, Waters Corp., Milford, MA, USA). Raw data (intensity) were normalized to pre-spiked internal standards. Fold changes (FC) were calculated as changes in peak intensity (compared to control) of each individual lipid, separately. For changes in composition (%), the sum intensity of a given class was used as 100%. No comparisons between different classes were made. Four independent biological replicates were included.

### 4.7. Determination of Starch

Pellets of TMBE extraction, which contain starch and proteins, were washed once in cold methanol to remove residual lipids and once in 0.2% Triton X100, 20 mM Tris-Cl, pH 7.5 solution to remove proteins. Pellets were then incubated for 12 h with 0.1 N NaOH followed by iodine staining, as previously described [38]. 

### 4.8. Statistical Analysis

All statistical calculations (% of total, fold-change) were done using one-way ANOVA, post-hoc LSD testing. Changes considered significant when *p* value < 0.05.

Error bars: if not stated differently, these represent standard deviation. The 7-day samples were excluded from all statistical analysis as these are composed of a duplicate only.

PCA plots include all replicates (not average) using normalized peak areas.

## 5. Conclusions

The dynamic changes in level and turnover of the FA pool imply that the supply of FA is the bottleneck for TAG biosynthesis under N deprivation in *D.* *tertiolecta*. This observation is consistent with previous studies in other algae and plants, demonstrating that supplementation of external FA during N deprivation enhances TAG biosynthesis and that free FA levels are elevated during stress conditions prior to TAG accumulation [37,69,70]. Based on these results, we propose that activation of TAG biosynthesis under N deprivation in *D. tertiolecta* proceeds in two kinetically distinct stages: In the initial phase of N deprivation, de novo FA biosynthesis is activated due to accelerated production of acetyl-CoA, which is assimilated into PL [38,65], in parallel to enhanced starch accumulation (Scheme 1, stage 1). In the second stage, during 12–120 h of N deprivation, FA incorporation into TAG proceeds partly via the Kennedy pathway and mostly via acyl transfer, involving PL intermediates, which is likely to involve DGDG, MGDG, and possibly PG in the chloroplast and PC in ER membranes (Summarized in Scheme 1, stage 2, see also [15]). These findings may have implications for future attempts to manipulate the production of TAG and of other high-value products from microalgae.

## Figures and Tables

**Table 1:** Composition of free FA in the control and in N-deprived total lipid extracts (%).

*FA*	*16:0*	*18:0*	*18:1*	*18:2*	*18:3*	*X*
+N	10.2 ± 0.2	9.0 ± 0.9	48.2 ± 5.0	2.3 ± 0.1	25.5 ± 1.9	4.8 ± 0.5
−N	10 ± 1.3	12.2 ± 2.1	70.4 ± 3.6	0	0	7.2 ± 0.5

FA compositions were calculated from peak areas of FA components shown in Figure 2 with reference to known FA standards (stearic—18:0, oleic—18:1, palmitic—16:0, linoleic—18:2, linolenic—18:3, X—not identified) and expressed as % of the total FA (means ± SD of 3 independent experiments, *n* = 3).

**Table 2:** Summary of ^14^C-PlA incorporation rates and steady-state concentrations of different lipids under 48 h control and N-deprived cultures.

Lipid	Incorporation Rate [f mol PlA × min^−1^ × 10^6^ cells^−1^]			Steady-State Concentration [n mol C 10^6^ cells^−1^]		
	+N	−N	−N/+N	+N	−N	−N/+N
*PC*	294 ± 30	184 ± 14	0.62 ± 0.13	2.6 ± 0.3	3.6 ± 0.2	1.38 ± 0.13
*FA*	58 ± 10	144 ± 14	2.48 ± 0.2	32 ± 4	8.8 ± 1	0.28 ± 0.17
*DAG*	142 ± 10	120 ± 10	0.85 ± 0.1	10.4 ± 1	16.1 ± 2	1.54 ± 0.16
*DGDG*	44 ± 6	92 ± 8	2.09 ± 0.16	54 ± 4	45 ± 6	0.83 ± 0.17
*SQDG + PG*	82 ± 4	92 ± 6	1.12 ± 0.08	22.4 ± 1.7	15.5 ± 1.1	0.69 ± 0.1
*TAG*	40 ± 2	188 ± 20	4.7 ± 0.09	2 ± 0.2	123 ± 3	6.1 ± 0.1

Initial flux rates were calculated from the incorporation of PlA into different lipids during the first 5 min (data taken from Figure 4). Steady-state concentrations were calculated from total ^14^C contents in different lipids after prolonged pre-labeling with ^14^C-bicarbonate (Figure 2B). +N, control cells cultured 48 h in complete growth medium; −N, cultures deprived of nitrogen for 48 h. ± standard deviations of 3 independent experiments (*n* = 3).

## Data Availability

All raw data are available in the online version.

## Supplementary Materials

The following are available online at https://www.mdpi.com/article/10.3390/md19070368/s1, Supplement File 1: Calculation and measurement of dilution of ^14^C-bicarbonate with atmospheric CO_2_; Supplement File 2: Quenching conditions for lipid extraction; Supplement File 3: Data—normalized peak area; Table S1: Quenching of ^14^C-PlA incorporation; Table S2: Time course of incorporation of ^14^C-PlA into lipids in complete and in N-deprived media; Table S3: A comparison of different quenching conditions for lipid extraction. Figure S1: Total lipidome separation by PCA. Figure S2: Steady-state levels of individual lipids during 24 h of N deprivation. Figure S3A: Does PlA influence TAG formation in D. tertiolecta?. Figure S3B: Competition-dilution of supplemented PlA with internal PlA pools. Figure S4: Competition-dilution of supplemented PlA with internal PlA pools. Figure S5: Incorporation of 14C-PlA into different lipids. Figure S6: Relative changes in the level and composition of major PL species.

## References

[1] Cakmak T., Angun P., Demiray Y.E., Ozkan A.D., Elibol Z., Tekinay T. (2012). Differential effects of nitrogen and sulfur deprivation on growth and biodiesel feedstock production of Chlamydomonas reinhardtii. Biotechnol. Bioeng..

[2] Kropat J., Hong-Hermesdorf A., Casero D., Ent P., Castruita M., Pellegrini M., Merchant S.S., Malasarn D. (2011). A revised mineral nutrient supplement increases biomass and growth rate in Chlamydomonas reinhardtii. Plant J..

[3] Goncalves E.C., Koh J., Zhu N., Yoo M., Chen S., Matsuo T., Johnson J. (2016). V Nitrogen starvation-induced accumulation of triacylglycerol in the green algae: Evidence for a role for ROC40, a transcription factor involved in circadian rhythm. Plant J..

[4] Napier J.A. (2007). The Production of Unusual Fatty Acids in Transgenic Plants. Annu. Rev. Plant Biol..

[5] Hu Q., Sommerfeld M., Jarvis E., Ghirardi M., Posewitz M., Seibert M., Darzins A. (2008). Microalgal triacylglycerols as feedstocks for biofuel production: Perspectives and advances. Plant J..

[6] Harwood J.L., Guschina I.A. (2009). The versatility of algae and their lipid metabolism. Biochimie.

[7] Work V.H., Radakovits R., Jinkerson R.E., Meuser J.E., Elliott L.G., Vinyard D.J., Laurens L.M.L., Dismukes G.C., Posewitz M.C. (2010). Increased lipid accumulation in the Chlamydomonas reinhardtii sta7-10 starchless isoamylase mutant and increased carbohydrate synthesis in complemented strains. Eukaryot. Cell.

[8] Johnson X., Alric J. (2012). Interaction between starch breakdown, acetate assimilation, and photosynthetic cyclic electron flow in Chlamydomonas reinhardtii. J. Biol. Chem..

[9] Matich E.K., Ghafari M., Camgoz E., Caliskan E., Pfeifer B.A., Haznedaroglu B.Z., Atilla-Gokcumen G.E. (2018). Time-series lipidomic analysis of the oleaginous green microalga species Ettlia oleoabundans under nutrient stress. Biotechnol. Biofuels.

[10] Li-Beisson Y., Shorrosh B., Beisson F., Andersson M.X., Arondel V., Bates P.D., Baud S., Bird D., DeBono A., Durrett T.P. (2010). Acyl-Lipid Metabolism. Arab. B..

[11] Fan J., Andre C., Xu C. (2011). A chloroplast pathway for the de novo biosynthesis of triacylglycerol in Chlamydomonas reinhardtii. FEBS Lett..

[12] Goodson C., Roth R., Wang Z.T., Goodenough U. (2011). Structural correlates of cytoplasmic and chloroplast lipid body synthesis in Chlamydomonas reinhardtii and stimulation of lipid body production with acetate boost. Eukaryot. Cell.

[13] Liu B., Benning C. (2013). Lipid metabolism in microalgae distinguishes itself. Curr. Opin. Biotechnol..

[14] Li-Beisson Y., Beisson F., Riekhof W. (2015). Metabolism of acyl-lipids in Chlamydomonas reinhardtii. Plant J..

[15] Pick U., Avidan O. (2017). Triacylglycerol is produced from starch and polar lipids in the green alga *Dunaliella tertiolecta*. J. Exp. Bot..

[16] Küpper F.C., Gaquerel E., Cosse A., Adas F., Peters A.F., Müller D.G., Kloareg B., Salaün J.-P., Potin P. (2009). Free Fatty Acids and Methyl Jasmonate Trigger Defense Reactions in Laminaria digitata. Plant Cell Physiol..

[17] Parrish C.C., deFreitas A.S.W., Bodennec G., Macpherson E.J., Ackman R.G. (1991). Lipid composition of the toxic marine diatom, Nitzschia pungens. Phytochemistry.

[18] Li N., Gügel I.L., Giavalisco P., Zeisler V., Schreiber L., Soll J., Philippar K. (2015). FAX1, a Novel Membrane Protein Mediating Plastid Fatty Acid Export. PLoS Biol..

[19] Zhang M., Fan J., Taylor D.C., Ohlrogge J.B. (2009). DGAT1 and PDAT1 acyltransferases have overlapping functions in Arabidopsis triacylglycerol biosynthesis and are essential for normal pollen and seed development. Plant Cell.

[20] Bates P.D., Stymne S., Ohlrogge J. (2013). Biochemical pathways in seed oil synthesis. Curr. Opin. Plant Biol..

[21] Miller R., Wu G., Deshpande R.R., Vieler A., Gartner K., Li X., Moellering E.R., Zauner S., Cornish A.J., Liu B. (2010). Changes in transcript abundance in chlamydomonas reinhardtii following nitrogen deprivation predict diversion of metabolism. Plant Physiol..

[22] Boyle N.R., Page M.D., Liu B., Blaby I.K., Casero D., Kropat J., Cokus S.J., Hong-Hermesdorf A., Shaw J., Karpowicz S.J. (2012). Three acyltransferases and nitrogen-responsive regulator are implicated in nitrogen starvation-induced triacylglycerol accumulation in Chlamydomonas. J. Biol. Chem..

[23] Msanne J., Xu D., Konda A.R., Casas-Mollano J.A., Awada T., Cahoon E.B., Cerutti H. (2012). Metabolic and gene expression changes triggered by nitrogen deprivation in the photoautotrophically grown microalgae Chlamydomonas reinhardtii and Coccomyxa sp. C-169. Phytochemistry.

[24] Ramanan R., Kim B.H., Cho D.H., Ko S.R., Oh H.M., Kim H.S. (2013). Lipid droplet synthesis is limited by acetate availability in starchless mutant of Chlamydomonas reinhardtii. FEBS Lett..

[25] Chen J.E., Smith A.G. (2012). A look at diacylglycerol acyltransferases (DGATs) in algae. J. Biotechnol..

[26] Goncalves E.C., Wilkie A.C., Kirst M., Rathinasabapathi B. (2016). Metabolic regulation of triacylglycerol accumulation in the green algae: Identification of potential targets for engineering to improve oil yield. Plant Biotechnol. J..

[27] Sharma N., Anderson M., Kumar A., Zhang Y., Michael E.M., Abrams S.R., Zaharia L.I., Taylor D.C., Fobert P.R. (2008). Transgenic increases in seed oil content are associated with the differential expression of novel Brassica-specific transcripts. BMC Genom..

[28] La Russa M., Bogen C., Uhmeyer A., Doebbe A., Filippone E., Kruse O., Mussgnug J.H. (2012). Functional analysis of three type-2 DGAT homologue genes for triacylglycerol production in the green microalga Chlamydomonas reinhardtii. J. Biotechnol..

[29] Niu Y.F., Zhang M.H., Li D.W., Yang W.D., Liu J.S., Bai W.B., Li H.Y. (2013). Improvement of neutral lipid and polyunsaturated fatty acid biosynthesis by overexpressing a type 2 diacylglycerol acyltransferase in marine diatom Phaeodactylum tricornutum. Mar. Drugs.

[30] Bates P.D., Browse J. (2012). The significance of different diacylgycerol synthesis pathways on plant oil composition and bioengineering. Front. Plant Sci..

[31] Guschina I.A., Everard J.D., Kinney A.J., Quant P.A., Harwood J.L. (2014). Studies on the regulation of lipid biosynthesis in plants: Application of control analysis to soybean. Biochim. Biophys. Acta Biomembr..

[32] Hurlock A.K., Roston R.L., Wang K., Benning C. (2014). Lipid trafficking in plant cells. Traffic.

[33] Park J.J., Wang H., Gargouri M., Deshpande R.R., Skepper J.N., Holguin F.O., Juergens M.T., Shachar-Hill Y., Hicks L.M., Gang D.R. (2015). The response of Chlamydomonas reinhardtii to nitrogen deprivation: A systems biology analysis. Plant J..

[34] Blaby I.K., Glaesener A.G., Mettler T., Fitz-Gibbon S.T., Gallaher S.D., Liu B., Boyle N.R., Kropat J., Stitt M., Johnson S. (2013). Systems-level analysis of nitrogen starvation-induced modifications of carbon metabolism in a Chlamydomonas reinhardtii starchless mutant. Plant Cell.

[35] Goodenough U., Blaby I., Casero D., Gallaher S.D., Goodson C., Johnson S., Lee J.H., Merchant S.S., Pellegrini M., Roth R. (2014). The path to triacylglyceride obesity in the sta6 strain of Chlamydomonas reinhardtii. Eukaryot. Cell.

[36] Siaut M., Cuiné S., Cagnon C., Fessler B., Nguyen M., Carrier P., Beyly A., Beisson F., Triantaphylidès C., Li-Beisson Y. (2011). Oil accumulation in the model green alga Chlamydomonas reinhardtii: Characterization, variability between common laboratory strains and relationship with starch reserves. BMC Biotechnol..

[37] Fan J., Yan C., Andre C., Shanklin J., Schwender J., Xu C. (2012). Oil accumulation is controlled by carbon precursor supply for fatty acid synthesis in Chlamydomonas reinhardtii. Plant Cell Physiol..

[38] Avidan O., Brandis A., Rogachev I., Pick U. (2015). Enhanced acetyl-CoA production is associated with increased triglyceride accumulation in the green alga Chlorella desiccata. J. Exp. Bot..

[39] Malitsky S., Ziv C., Rosenwasser S., Zheng S., Schatz D., Porat Z., Ben-Dor S., Aharoni A., Vardi A. (2016). Viral infection of the marine alga Emiliania huxleyi triggers lipidome remodeling and induces the production of highly saturated triacylglycerol. New Phytol..

[40] Martin G.J.O., Hill D.R.A., Olmstead I.L.D., Bergamin A., Shears M.J., Dias D.A., Kentish S.E., Scales P.J., Botté C.Y., Callahan D.L. (2014). Lipid profile remodeling in response to nitrogen deprivation in the microalgae Chlorella sp. (Trebouxiophyceae) and Nannochloropsis sp. (Eustigmatophyceae). PLoS ONE.

[41] Han D., Jia J., Li J., Sommerfeld M., Xu J., Hu Q. (2017). Metabolic remodeling of membrane glycerolipids in the microalga Nannochloropsis oceanica under nitrogen deprivation. Front. Mar. Sci..

[42] Li-Beisson Y., Thelen J.J., Fedosejevs E., Harwood J.L. (2019). The lipid biochemistry of eukaryotic algae. Prog. Lipid Res..

[43] Li X., Moellering E.R., Liu B., Johnny C., Fedewa M., Sears B.B., Kuo M.H., Benning C. (2012). A galactoglycerolipid lipase is required for triacylglycerol accumulation and survival following nitrogen deprivation in chlamydomonas reinhardtiic w. Plant Cell.

[44] Davidi L., Shimoni E., Khozin-Goldberg I., Zamir A., Pick U. (2014). Origin of β-carotene-rich plastoglobuli in Dunaliella bardawil. Plant Physiol..

[45] Cronan J.E. (1982). Molecular Properties of Short Chain Acyl Thioesters of Acyl Carrier Protein*. J. Biol. Chem..

[46] Davidi L., Katz A., Pick U. (2012). Characterization of major lipid droplet proteins from Dunaliella. Planta.

[47] Ho Cho S., Thompson G.A. (1987). On the Metabolic Relationships between Monogalactosyldiacylglycerol and Digalactosyldiacylglycerol Molecular Species in Dunaliella salina. J. Biol. Chem..

[48] Bates P.D., Durrett T.P., Ohlrogge J.B., Pollard M. (2009). Analysis of acyl fluxes through multiple pathways of triacylglycerol synthesis in developing soybean embryos. Plant Physiol..

[49] Liping W., Shen W., Kazachkov M., Chen G., Chen Q., Carlsson A.S., Stymne S., Weselake R.J., Zou J. (2012). Metabolic interactions between the lands cycle and the kennedy pathway of glycerolipid synthesis in arabidopsis developing seedsw. Plant Cell.

[50] Allen D.K., Bates P.D., Tjellström H. (2015). Tracking the metabolic pulse of plant lipid production with isotopic labeling and flux analyses: Past, present and future. Prog. Lipid Res..

[51] Bates P.D., Ohlrogge J.B., Pollard M. (2007). Incorporation of newly synthesized fatty acids into cytosolic glycerolipids in pea leaves occurs via acyl editing. J. Biol. Chem..

[52] Scrimgeour C.M., Harwood J.L. (2007). The Lipidomic Handbook.

[53] Zienkiewicz K., Du Z.Y., Ma W., Vollheyde K., Benning C. (2016). Stress-induced neutral lipid biosynthesis in microalgae—Molecular, cellular and physiological insights. Biochim. Biophys. Acta Mol. Cell Biol. Lipids.

[54] Guschina I.A., Harwood J.L. (2006). Lipids and lipid metabolism in eukaryotic algae. Prog. Lipid Res..

[55] Lippold F., vom Dorp K., Abraham M., Hölzl G., Wewer V., Yilmaz J.L., Lager I., Montandon C., Besagni C., Kessler F. (2012). Fatty acid phytyl ester synthesis in chloroplasts of Arabidopsis. Plant Cell.

[56] Wang K., Froehlich J.E., Zienkiewicz A., Hersh H.L., Benning C. (2017). A plastid phosphatidylglycerol lipase contributes to the export of acyl groups from plastids for seed oil biosynthesis. Plant Cell.

[57] Davidi L., Levin Y., Ben-Dor S., Pick U. (2015). Proteome analysis of cytoplasmatic and plastidic β-carotene lipid droplets in Dunaliella bardawil1[OPEN]. Plant Physiol..

[58] Dahlqvist A., Ståhl U., Lenman M., Banas A., Lee M., Sandager L., Ronne H., Stymne S. (2000). Phospholipid:diacylglycerol acyltransferase: An enzyme that catalyzes the acyl-CoA-independent formation of triacylglycerol in yeast and plants. Proc. Natl. Acad. Sci. USA.

[59] Yoon K., Han D., Li Y., Sommerfeld M., Hu Q. (2012). Phospholipid:Diacylglycerol acyltransferase is a multifunctional enzyme involved in membrane lipid turnover and degradation while synthesizing triacylglycerol in the unicellular green microalga chlamydomonas reinhardtii. Plant Cell.

[60] Allen J.W., DiRusso C.C., Black P.N. (2015). Triacylglycerol synthesis during nitrogen stress involves the prokaryotic lipid synthesis pathway and acyl chain remodeling in the microalgae Coccomyxa subellipsoidea. Algal Res..

[61] Sakurai K., Mori N., Sato N. (2014). Detection and characterization of phosphatidylcholine in various strains of the genus Chlamydomonas (Volvocales, Chlorophyceae). J. Plant Res..

[62] Kim S.H., Liu K.H., Lee S.Y., Hong S.J., Cho B.K., Lee H., Lee C.G., Choi H.K. (2013). Effects of Light Intensity and Nitrogen Starvation on Glycerolipid, Glycerophospholipid, and Carotenoid Composition in *Dunaliella tertiolecta* Culture. PLoS ONE.

[63] Du Z.Y., Lucker B.F., Zienkiewicz K., Miller T.E., Zienkiewicz A., Sears B.B., Kramer D.M., Benning C. (2018). Galactoglycerolipid lipase PGD1 is involved in thylakoid membrane remodeling in response to adverse environmental conditions in chlamydomonas. Plant Cell.

[64] Moellering E.R., Benning C. (2011). Galactoglycerolipid metabolism under stress: A time for remodeling. Trends Plant Sci..

[65] Avidan O., Pick U. (2015). Acetyl-CoA synthetase is activated as part of the PDHbypass in the oleaginous green alga Chlorella desiccata. J. Exp. Bot..

[66] Bates P.D., Fatihi A., Snapp A.R., Carlsson A.S., Browse J., Lu C. (2012). Acyl editing and headgroup exchange are the major mechanisms that direct polyunsaturated fatty acid flux into triacylglycerols. Plant Physiol..

[67] Ben-Amotz A., Shaish A., Avron M. (1989). Mode of Action of the Massively Accumulated β-Carotene of Dunaliella bardawil in Protecting the Alga against Damage by Excess Irradiation. Plant Physiol..

[68] Zalogin T.R., Pick U. (2014). Azide improves triglyceride yield in microalgae. Algal Res..

[69] Dakhma W.S., Zarrouk M., Cherif A. (1995). Effects of drought-stress on lipids in rape leaves. Phytochemistry.

[70] Upchurch R.G. (2008). Fatty acid unsaturation, mobilization, and regulation in the response of plants to stress. Biotechnol. Lett..

