# Supplementary material for: Fatty Acid Production and Direct Acyl Transfer through Polar Lipids Control TAG Biosynthesis during Nitrogen Deprivation in the Halotolerant Alga Dunaliella tertiolecta
Source: Mar Drugs. 2021 Jun 25;19(7):368. doi: 10.3390/md19070368 (PMC8304655; doi:10.3390/md19070368)
Supplement: Supplementary file 1 [file marinedrugs-19-00368-s001.zip › Figure S1.pdf]

Figure S1:

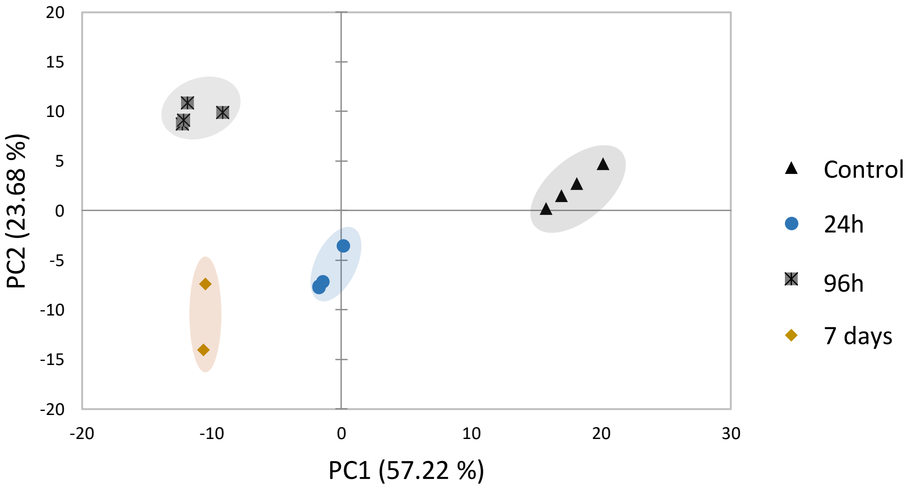

Figure S1: Total lipidome separation by PCA (including TAG). PCA explains 80% of the distribution of the data, and shows a clear separation between induced and non-induced cells suggesting redistribution of the lipidome in response to N deprivation. Data used – normalized peak area.
