# Supplementary material for: Fatty Acid Production and Direct Acyl Transfer through Polar Lipids Control TAG Biosynthesis during Nitrogen Deprivation in the Halotolerant Alga Dunaliella tertiolecta
Source: Mar Drugs. 2021 Jun 25;19(7):368. doi: 10.3390/md19070368 (PMC8304655; doi:10.3390/md19070368)
Supplement: Supplementary file 1 [file marinedrugs-19-00368-s001.zip › Figure S2.pdf]

Figure S2:

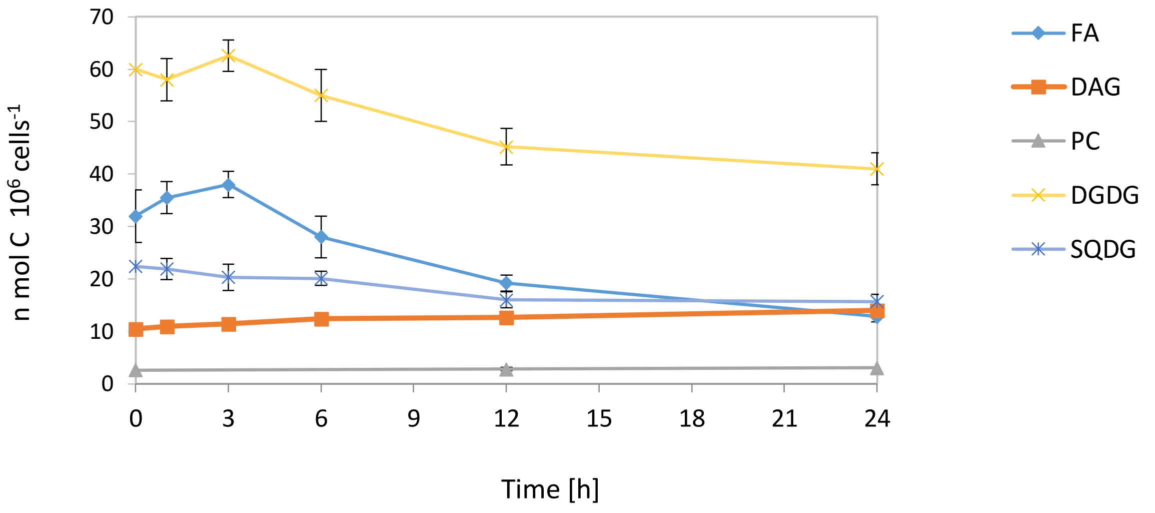

Fig. S2: Extended view of changes in the steady-state levels of individual lipids during 24 h of N deprivation (taken from Figure 1B). Error bars represent standard deviation (n=3).
