# Supplementary material for: Fatty Acid Production and Direct Acyl Transfer through Polar Lipids Control TAG Biosynthesis during Nitrogen Deprivation in the Halotolerant Alga Dunaliella tertiolecta
Source: Mar Drugs. 2021 Jun 25;19(7):368. doi: 10.3390/md19070368 (PMC8304655; doi:10.3390/md19070368)
Supplement: Supplementary file 1 [file marinedrugs-19-00368-s001.zip › Figure S3.pdf]

Figure S3:

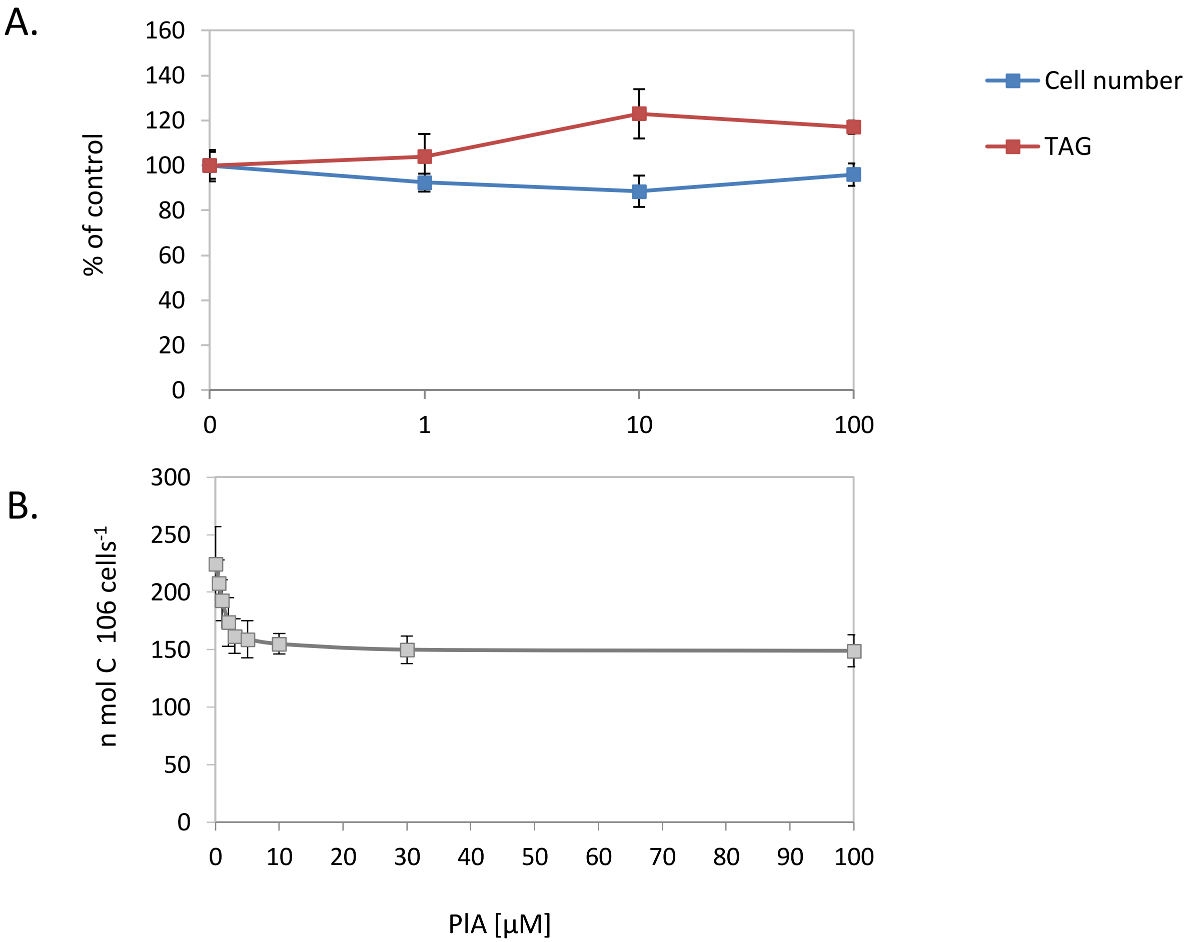

Fig. S3A: Does PlA influence TAG formation in *D. tertiolecta*? *D. tertiolecta* cells were cultured for 4 days in N-deficient media in the absence or presence of the indicated concentrations of PlA. TAG level was measured by the iodine method. Results are expressed as % of control values (- PlA). The control (100%) values represent 1.3 x 10^6 cells^{-1}, 0.75 ± 0.05 µg TAG · 10^6 cells^{-1}.

Fig. S3B: Competition-dilution of supplemented PlA with internal PlA pools. *D. tertiolecta* cells were cultured for 24 h in complete growth medium in the presence of 50 mM ^{14}C-bicarbonate and PlA at the indicated concentrations. At the end of the incubation lipids were extracted and the level of incorporation of ^{14}C into the total lipid fraction was determined. Results are expressed in n mol C 10^6 · cells^{-1} and represent 3 independent experiments. PlA - palmitic acid.
error bars represent standard deviation.
