# Supplementary material for: Fatty Acid Production and Direct Acyl Transfer through Polar Lipids Control TAG Biosynthesis during Nitrogen Deprivation in the Halotolerant Alga Dunaliella tertiolecta
Source: Mar Drugs. 2021 Jun 25;19(7):368. doi: 10.3390/md19070368 (PMC8304655; doi:10.3390/md19070368)

Figure S4:

A.

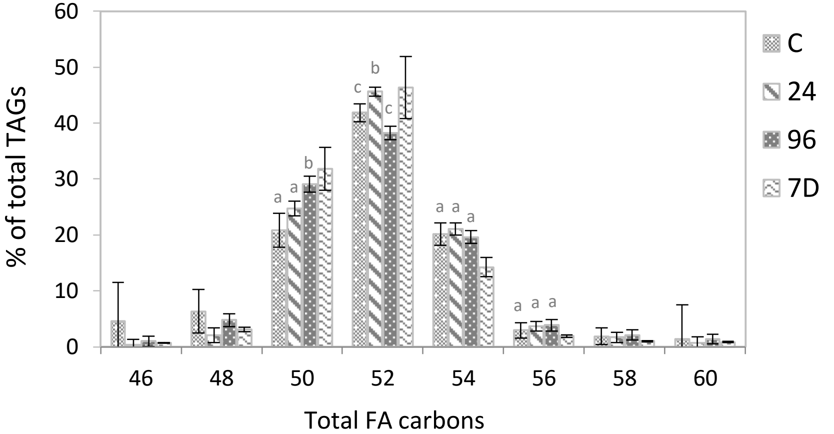

Figure S4: TAG accumulation following N deprivation. (A) Changes in composition of TAG in control and N-deprived cells (% of total), showing a clear preference for 50-54C TAG synthesis. The X axis represents the sum of carbons of 3 FAs attached to a glycerol backbone. Letters represent statistical tests within each TAG class. (B) Relative changes in the level (bottom) and composition (up) of major TAG species. Data are presented as fold-change (relative to control) and as % of total, respectively. Error bars: for %, represent standard deviation, whereas in fold change calculations, error bars represent coefficient of variation. Letters indicate statistically significant changes. Statistical tests were carried out using ANOVA, Post Hoc LSD test, $p<0.05$.

B.

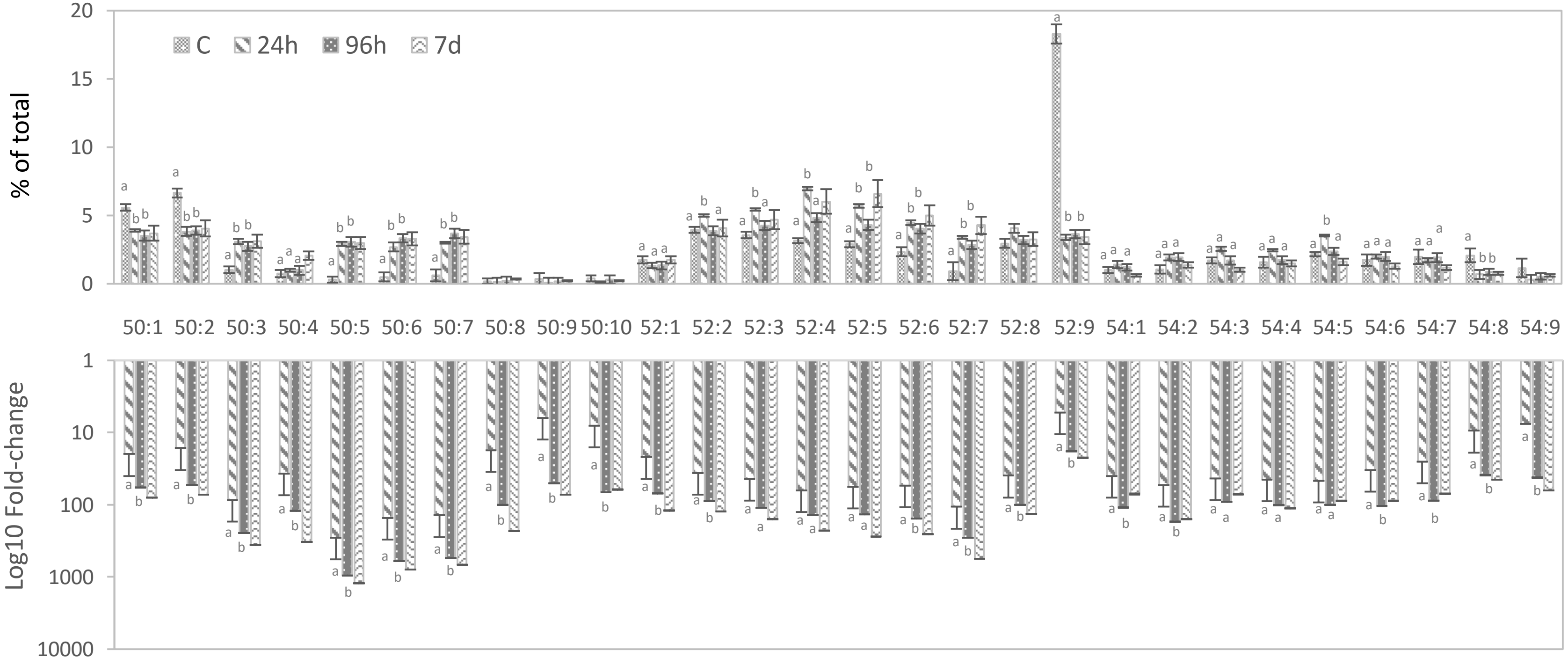

Supplement: Supplementary file 1 [file marinedrugs-19-00368-s001.zip › Figure S4.pdf]
