# Supplementary material for: Fatty Acid Production and Direct Acyl Transfer through Polar Lipids Control TAG Biosynthesis during Nitrogen Deprivation in the Halotolerant Alga Dunaliella tertiolecta
Source: Mar Drugs. 2021 Jun 25;19(7):368. doi: 10.3390/md19070368 (PMC8304655; doi:10.3390/md19070368)
Supplement: Supplementary file 1 [file marinedrugs-19-00368-s001.zip › Figure S5.pdf]

Figure S5:

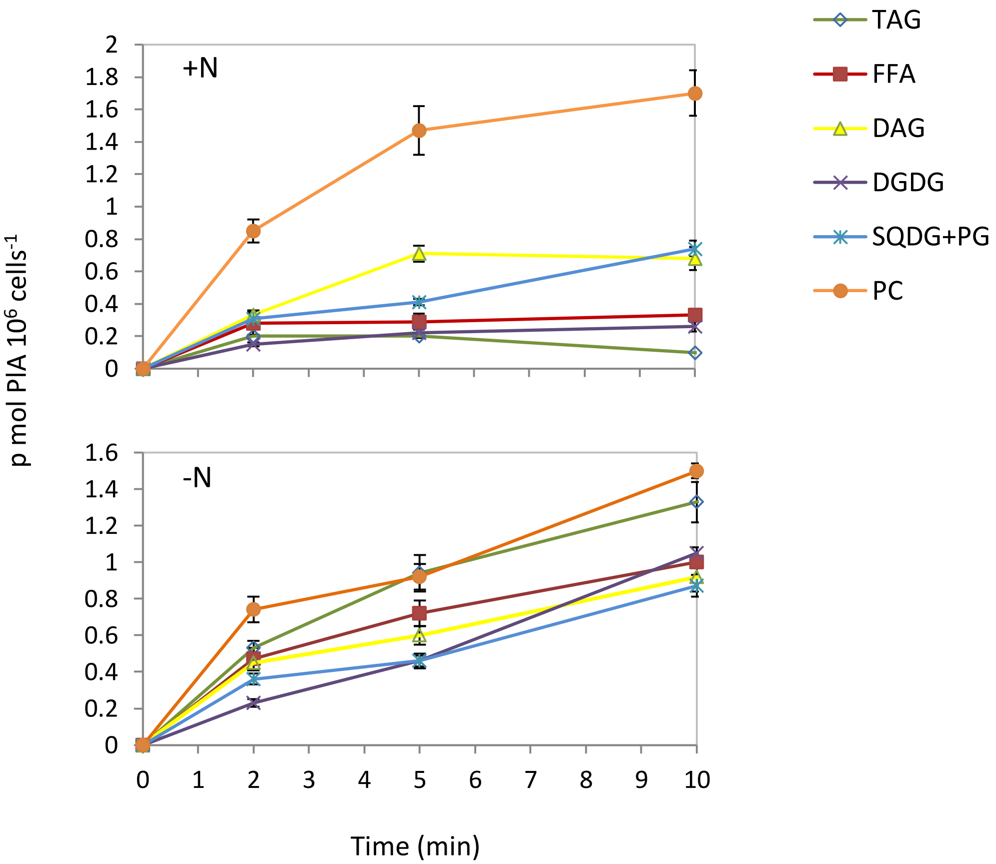

Figure S5: Extended view of incorporation of ^{14}C-PIA into different lipids. *D. tertiolecta* during the first 10 minutes (taken from Fig. 4). Error bars represent standard deviation.
