# Supplementary material for: Fatty Acid Production and Direct Acyl Transfer through Polar Lipids Control TAG Biosynthesis during Nitrogen Deprivation in the Halotolerant Alga Dunaliella tertiolecta
Source: Mar Drugs. 2021 Jun 25;19(7):368. doi: 10.3390/md19070368 (PMC8304655; doi:10.3390/md19070368)
Supplement: Supplementary file 1 [file marinedrugs-19-00368-s001.zip › Figure S6.pdf]

Figure S6

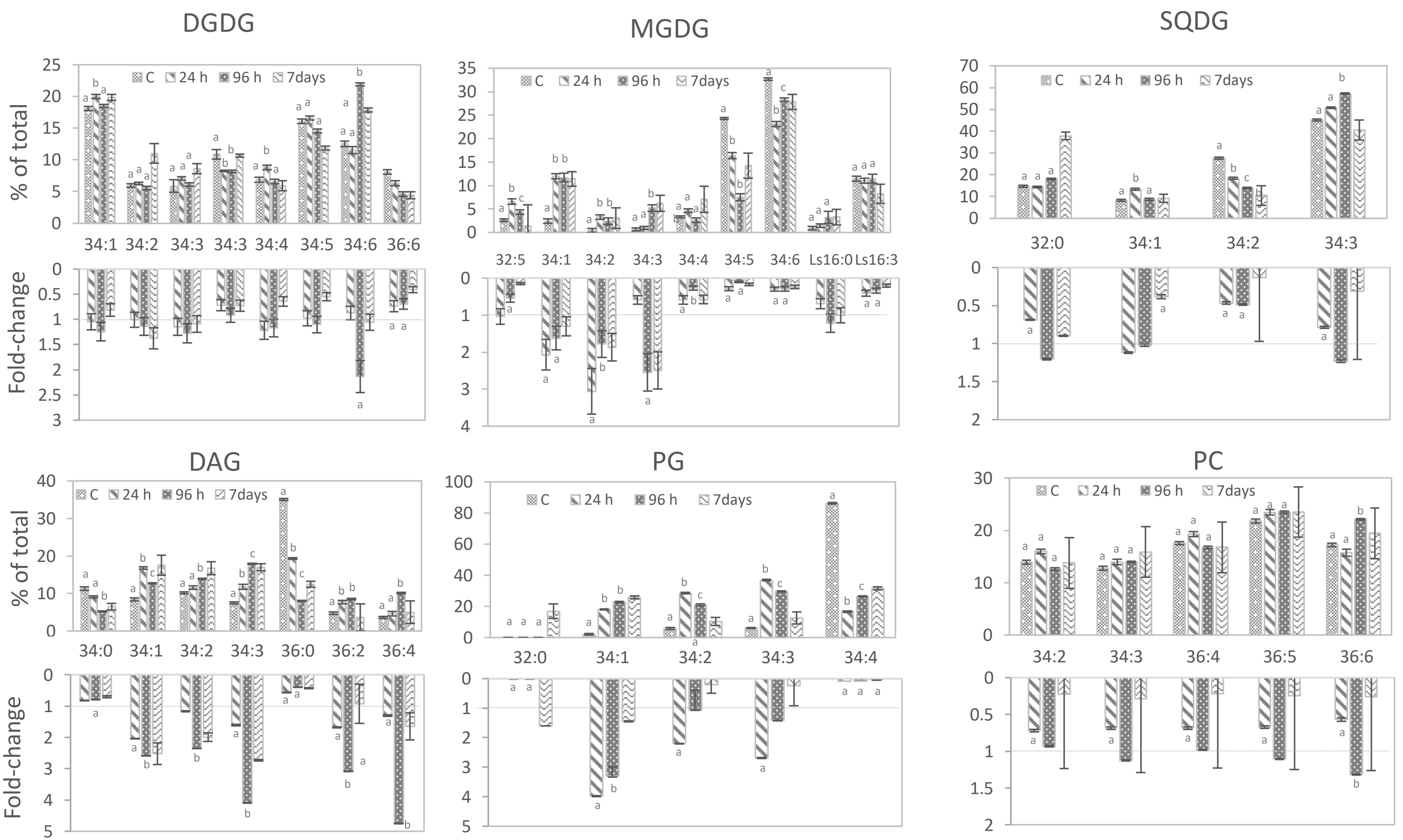

Figure S6: Relative changes in the level (bottom) and composition (up) of major PL species (polar lipids). Data presented for each individual lipid as fold-change (relative to control) and as % of total (class), respectively. Error bars: for % – standard deviation, for Fold change - coefficient of variation. Letters represent statistical tests within each class which were carried out using ANOVA, Post Hoc LSD test, p<0.05.

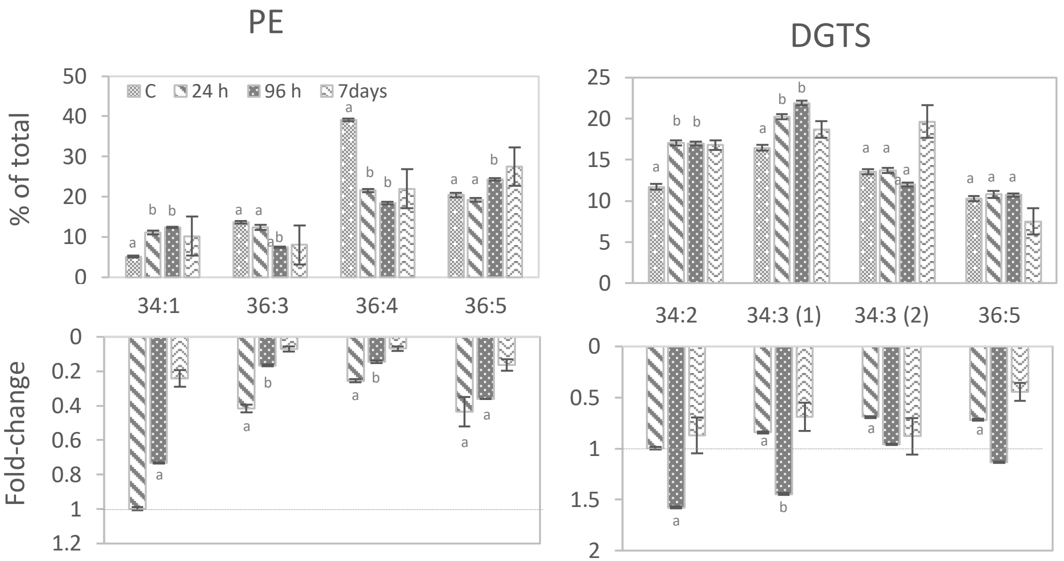
PE
DGTS
% of total
Fold-change
C
24 h
96 h
7days
34:1
36:3
36:4
36:5
34:2
34:3 (1)
34:3 (2)
36:5
