# Supplementary material for: Fatty Acid Production and Direct Acyl Transfer through Polar Lipids Control TAG Biosynthesis during Nitrogen Deprivation in the Halotolerant Alga Dunaliella tertiolecta
Source: Mar Drugs. 2021 Jun 25;19(7):368. doi: 10.3390/md19070368 (PMC8304655; doi:10.3390/md19070368)
Supplement: Supplementary file 1 [file marinedrugs-19-00368-s001.zip › Supplement 1.pdf]

Supplement 1: Calculation and measurement of dilution of ^{14}C-bicarbonate with atmospheric CO_2.

The dilution of ^{14}C-bicarbonate during the labeling by atmospheric CO_2 (Figure 2) was estimated by calculation and by direct measurements. Calculation of the level of CO_2 dissolution from the air was made by assuming a CO_2 transfer velocity of 10 cm h^{-1} in air (46) and a CO_2 concentration of 400 ppm. The calculated level of CO_2 dissolution in 24 h is 430 µM which is less than 1% of the total bicarbonate in the labeling solution. For the experimental measurement of CO_2 dissolution, 400 ppm of ^{14}C-CO_2 gas was injected though a syringe to a flask sealed with a rubber crock, in which *D. tertiolecta* cells grow in complete growth medium containing 50 mM unlabeled bicarbonate and incubated for 24 h under the experimental conditions described above. The measured level of dissolved ^{14}C-CO_2 was 580 µM. In parallel, we measured the loss of ^{14}C from the ^{14}C-bicarbonate labeling medium due to diffusion and the level of ^{14}C incorporation into the cells during the first 24 h of incubation (14), and obtained values of 6% and 3% of the total ^{14}C-bicarbonate, respectively. The calculated dilution factor of ^{14}C in the labeling medium by atmospheric CO_2 according to these values is 1.013 (1.3%). It should be noted that the significance of dilution by atmospheric CO_2 is relevant only during the first 48 h of labeling because under N deprivation photosynthetic CO_2 assimilation decreases to 50% after 24 h and to less than 10% after 48 h. After this period, lipids are produced from the pre-labeled starch and PL [15].
