# Supplementary material for: Fatty Acid Production and Direct Acyl Transfer through Polar Lipids Control TAG Biosynthesis during Nitrogen Deprivation in the Halotolerant Alga Dunaliella tertiolecta
Source: Mar Drugs. 2021 Jun 25;19(7):368. doi: 10.3390/md19070368 (PMC8304655; doi:10.3390/md19070368)
Supplement: Supplementary file 1 [file marinedrugs-19-00368-s001.zip › Supplement 2.pdf]

Supplement 2: Quenching conditions for lipid extraction.

The high levels of FFA pool that is comparable to other major lipid components (Figures 2B, 2C), is surprising because FFA concentrations in plants and in algae are expected to be very low, except for some diatoms [17]. A high level of FFA in lipid extracts could result from activation of lipases before or during lipid extraction. Therefore, we checked if the FFA pool may result from hydrolysis of lipids by lipases, by comparing different quenching treatments that are known to inhibit lipases (DMSO, *iso*-propanol or trichloroacetic acid; [17]) at different temperatures (25-90 °C), and by using different extraction techniques (methanol, chloroform:methanol or methyl-*tert*butylether). We obtained essentially the same levels of FA (Supplement 3, Table S3), indicating that there is no significant hydrolysis of lipids by lipases during the extraction. Because FA, like palmitic acid (PlA), rapidly equilibrate across Dunaliella cell membranes, a BSA treatment is expected to effectively deplete the internal FFA pool. Nevertheless, including BSA in the quenching solution prior to cell lysis did not significantly affect the FFA pool size (Table S3). According to these considerations, the FFA pool that we measure does not result from hydrolysis of lipids.

S3: Table S3: A comparison of different quenching conditions for lipid extraction.

**Quenching conditions**	**DPM in FA band**
DMSO, no heating	1,300 ± 220
DMSO, heating to 70 °C	1,770 ± 310
DMSO, heating to 90 °C	1,469 ± 120
Iso-propanol, heating to 90 °C	1,510 ± 185
DMSO+2 mL MeOH+ 1 mL CHCl3, no heating	1,540 ± 200
10% trichloroacetic acid	1,680 ± 155
BSA treatment, DMSO, 70 °C	1,420 ± 120

D. tertiolecta cultures were incubated with ^{14}C bicarbonate in complete growth medium for 48 h, as described under Experimental Procedures. Samples were washed twice the pellets were quenched by addition of 200 µL quenching solution and by heating as specified in the table. BSA treatment: before the second wash, cells were incubated for 10’ with 1% defatted BSA at 4 °C. Lipids were extracted, resolved on TLC plates, the FA bands were cut out, extracted and their ^{14}C content was determined. Results are expressed in disintegrations per min (DPM) and represent 3 separate experiments.
